# Orchestrating the proteome with post-translational modifications

**DOI:** 10.1093/jxb/ery295

**Published:** 2018-08-31

**Authors:** Steven H Spoel

**Affiliations:** Institute of Molecular Plant Sciences, School of Biological Sciences, University of Edinburgh, Edinburgh EH9 3BF, United Kingdom

**Keywords:** Phenotypic traits, phosphorylation, post-translational modification, protein cleavage, proteome, redox-based modifications, SUMOylation, translation, ubiquitination

**Post-translational modifications (PTMs) add tremendous complexity to cellular proteomes. The large variety of these modifications and their concurrent appearance in proteins dramatically increase the proteome size from mere thousands to the order of millions of possible protein forms. Emerging evidence from plants indicates that PTMs intimately regulate numerous developmental programmes and responses to the environment. Reviews and research papers in this issue highlight our advanced knowledge of mechanisms that underpin several key PTMs and how these generate phenotypic traits that may be exploited to boost crop production.**

Cellular and molecular biology have historically focused on how the genetic code influences all aspects of life, including developmental traits and cellular or organismal responses to the changing environment. Conventional genetics and newly emerging genomic techniques have revealed that genomes harbour much more complex information than simply revealed by the number of genes they encode. In addition to gene transcription, post-transcriptional mechanisms play important roles in defining the functional output of the genome. Non-coding RNAs expressed from selected genomic regions regulate gene expression and protein translation, while pre-RNA processing generates further complexity in genomes with a relatively small number of genes (Licatalosi and Darnell, 2010). These advances in our understanding of how genomes function begin to reveal how a relatively small genome can generate highly sophisticated organismal outputs. In terms of complexity in functional outputs from any single gene, however, by far the largest amount of complexity is generated by diverse post-translational modifications (PTMs). The sheer number of possible PTMs combined with the multiple amino acid residues they occur on leads to nearly incomprehensible numbers of possible protein forms that could each support different functions (Box 1).

**Box 1. The diversity and complexity of post-translational modifications**

A subset of well-recognized post-translational modifications are shown and classed in categories based on type of modification. Chemical modifications are reversible and include phosphorylation (P), acetylation (Ac), methylation (Me), and redox-based modifications ranging from *S*-nitrosylation (SNO), thiolation (S–S), *S*-sulfenation (SOH) and *S*-sulfination (SO_2_H). Polypeptide modifications are also enzymatically reversible and include ubiquitination (Ub), SUMOylation (S), and other modifications by ubiquitin-like (Ubl) polypeptides. Modifications by complex molecules are reversible and include glycosylation, attachment of lipids (e.g. acylation, prenylation), ADP-ribosylation (Ri-ADP) and AMPylation (AMP). Finally, modifications of amino acids (asterisk) or of the polypeptide backbone are irreversible, including by deamidation, eliminylation and cleavage by proteolysis.

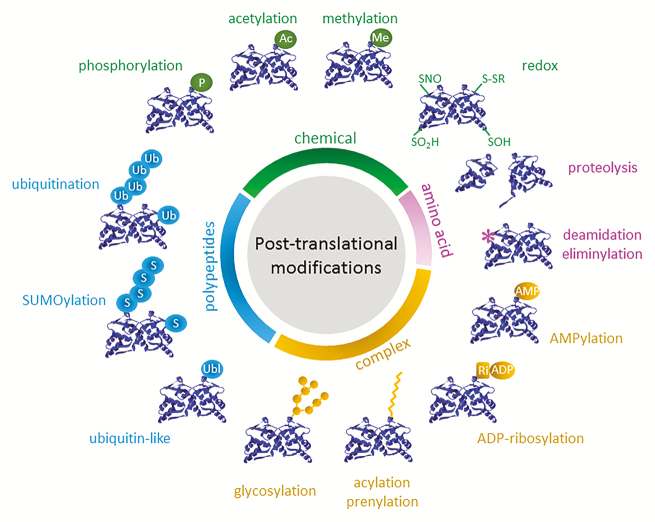

PTMs come in various forms, including the addition of chemical groups (e.g. phosphorylation, acetylation, methylation, redox-based modifications), addition of polypeptides (e.g. ubiquitination, SUMOylation and other ubiquitin-like protein conjugation), addition of complex molecules (e.g. acylation and prenylation, glycosylation, ADP-ribosylation, AMPylation), direct modification of amino acids (e.g. deamidation, eliminylation), and protein cleavage through a variety of proteolytic mechanisms (Box 1). Whereas modification of the protein backbone is irreversible, the addition of chemical groups, polypeptides and complex molecules is often reversible. Consequently, cells often employ PTMs to enable rapid and dynamic signalling by activating enzymes that function as ‘writers’ and ‘erasers’ of PTMs. Indeed, enzymes that regulate PTMs are vast in number and their fine-tuning allows cells to control the location and duration of PTM-based functional outputs. For example, in Arabidopsis phosphorylation is regulated by more than 1100 genes encoding protein kinases and phosphatases (Wang *et al.*, 2007), whereas over 1600 genes encode ubiquitin ligases and deubiquitinases (Mazzucotelli *et al.*, 2006; Yang *et al.*, 2007; Vierstra, 2009). Thus, a significant amount of the genome is dedicated to regulating the addition and removal of PTMs, reflecting their importance and functional versatility. Nonetheless, non-enzymatic processes also regulate PTMs. Most redox-based PTMs, including cysteine modifications by *S*-nitrosylation (–SNO), *S*-sulfenation (–SOH), *S*-thiolation (S–S), and *S*-sulfination (–SO_2_H) are thought to be predominantly generated by spontaneous reactivity with reactive oxygen or nitrogen species (Waszczak *et al.*, 2015). However, the removal of these redox-based modifications is often subject to enzymatic control by the superfamily of thioredoxin enzymes (Sevilla *et al.*, 2015; Mata-Pérez and Spoel, 2019).

The advent of increasingly powerful proteomic techniques, together with newly emerging molecular biology tools for the purification and specific detection of PTMs, has propelled research on PTMs in plant biology. The importance of PTMs is increasingly recognized but how they collectively orchestrate the proteome to yield different phenotypes remains largely unknown. The research papers and reviews in this special issue address this challenge head-on and provide new ways of thinking about the functions of PTMs at the interface between proteome and phenotype.

## Dynamic control of the proteome

To thrive as sessile organisms, plants have to cope with a large variety of biotic and abiotic stresses encountered in their ever-changing environment. With the genome largely fixed, dynamicity in proteome outputs is critical for responding rapidly and efficiently to emerging threats. Protein phosphorylation is arguably the best-studied PTM in providing dynamicity to proteomes (Gelens and Saurin, 2018). Its discovery early on in the 20th century heralded decades of future research into phosphoproteomes. Two studies are presented in this special issue that focus on identifying dynamic changes in plant protein phosphorylation in response to abiotic stresses. Nikonorova *et al.* (2018) catalogue temporal changes in phosphorylation after exposure to osmotic stress. Importantly, by revealing the dynamic phoshoproteome the authors were able to genetically pinpoint novel regulators of leaf growth under osmotic stress. Thus, proteomic characterization of dynamic PTMs has the potential to be a blueprint for identifying key regulatory signalling hubs that can subsequently be verified using conventional genetics. In the second study, Vu *et al.* (2018) identify changes in protein phosphorylation upon temperature shifting. Not only do they discover distinct phosphoproteome changes within different organs of wheat, but they also report on a rarely described phenomenon known as phospho-site interconversion. In this process phosphorylation is toggled between two neighbouring residues upon perception of a signal. Signal-induced interconversion of protein phosphoforms is likely to play an important role in temperature sensing and has the potential to dynamically regulate protein signalling and activity.

## Multitasking the proteome

Another feature of PTMs is that they fulfil a multitude of different functions in proteins that harbour them. The addition or removal of PTMs regulates enzyme activity, intra- and intermolecular changes in protein conformation, cellular localization, and interactions with protein partners or other biomolecules. Extraordinarily, a single type of PTM can be responsible for several of these different functions, depending on the site of modification and the context of the signalling pathway. This is particularly well exemplified by ubiquitin signalling, in which the highly conserved small polypeptide ubiquitin is attached to lysine residues of target proteins. Proteins may be modified by a single ubiquitin polypeptide (i.e. monoubiquitination) or at multiple different positions (multi-monoubiquitination). Moreover, seven internal lysine residues allow ubiquitin to form homogenous or heterogenous chains that in some cases are branched. These vastly different forms of ubiquitination allow this PTM to signal for a wide variety of processes that range from cellular signalling and protein sorting to targeted proteolysis (Komander and Rape, 2012). Miricescu *et al.* (2018) review how the multi-functionality of ubiquitin integrates environmental signals, illustrating that this PTM in all its different forms plays a central role in nearly all pathways regulated by developmental and stress hormones. Emerging evidence also indicates that ubiquitin modifications orchestrate cross talk between synergistic and antagonistic hormone-signalling pathways, allowing plants to fine tune cellular responses to their environment. The complexity provided by this PTM alone may explain why so much of each plant genome is devoted to encoding genes related to ubiquitin signalling.

Plant hormone perception represents a particularly intriguing multifunctional role for ubiquitin in the reprogramming of gene expression. Both Adams and Spoel (2018) and Miricescu *et al.* (2018) discuss how several plant hormones are perceived at the chromatin by ubiquitin E3 ligases. These E3 ligases multitask by functioning as direct hormone receptors and simultaneously as transcriptional cofactors. Multitasking is enabled by hormones acting as a molecular glue between the E3 ligase and its substrate, which is either a transcriptional coactivator or corepressor. The resulting hormone-induced ubiquitination of transcriptional co-regulators alters their intrinsic properties or targets them for degradation (Kelley and Estelle, 2012; Furniss and Spoel, 2015). Notably, elucidating the mechanisms by which these chromatin-associated E3 ligases multitask has far-reaching implications beyond plant biology. The biomedical sciences have vested interests in the design of therapeutic agents that mimic small molecule perception by E3 ligases, as dysfunction in ubiquitin signalling underpins many pathophysiological conditions in humans, including cancer, neurological disorders and immunodeficiency. Consequently, pharmacological drug discovery approaches are now being inspired by the mechanisms of plant hormone perception (Shabek and Zheng, 2014). By studying this natural process in plants we can contribute in unexpected ways to advances in biomedicine.

## Exploiting PTMs in crop biotechnology

While the discovery of new PTMs continues at a steady pace, new directions of research are beginning to ask how we can exploit the wide variety of PTMs to benefit advances in crop production. Over the past decades crop biotechnology approaches have predominantly taken single- or multi-gene knockout and overexpression approaches. While this has delivered several key advances, these are relatively brute force approaches that often lead to undesired trade-offs between growth and stress resilience. By comparison, editing of post-translationally modified sites or of the regulatory enzymes that control them could announce a more nuanced era in crop biotechnology. A number of the articles in this special issue explore how progress in our understanding of SUMOylation can contribute to biotechnological developments. The small polypeptide SUMO is related to ubiquitin but has distinct signalling roles that regulate both developmental and environmental responses. Like ubiquitin, protein SUMOylation is fine-tuned by a set of dedicated ligases and proteases. Benlloch and Lois (2018) provide insights into the important role of SUMOylation in abiotic stress responses. Particularly, heat and drought stress induce the accumulation of SUMO conjugates in various plant species, and genetic evidence indicates that SUMOylation controls plant fitness during times of stress. Thus, the SUMOylation machinery and its targets appear to be excellent objectives for next-generation crop improvement strategies.

While much of our fundamental knowledge of PTMs is gained from model plants, Garrido and Sadanandom (2018) argue that there is strong conservation across the plant kingdom in the regulatory mechanisms of SUMOylation. By searching the genomes of crop species they show that cereals in particular harbour comparatively large families of SUMO proteases, a group of enzymes capable of erasing SUMO modifications. As SUMO conjugation is carried out by a very limited number of ligases, they propose that SUMO proteases may introduce specificity into SUMO signalling pathways. Specificity provided by enzymes that ‘erase’ rather than ‘write’ PTMs has also been reported for redox signalling (Lemaire *et al.*, 2007; Kneeshaw *et al.*, 2014; Mata-Pérez and Spoel, 2019). Here only subsets of redox-based modifications are removed by selected thioredoxins that are tightly associated with specific signalling pathways. The factors that determine the selectivity of thioredoxins and other PTM erasers such as SUMO proteases remains poorly understood. Nonetheless, these enzymes are potentially high-value assets for crop breeding as their utilization could minimize the occurrence of unwanted pleiotropic effects often associated with conventional genetic editing strategies.

The promise of SUMO proteases for crop improvement is further highlighted by Castro *et al.* (2018*a*) who have uncovered a new subfamily of these enzymes that regulate growth and development, flowering time, seed size and yield. Given the importance of SUMO proteases, a further Viewpoint in the eXtra Botany section, by Castro *et al.* (2018*b*), reports on a community-wide effort to revise the nomenclature of a subgroup of these enzymes based on a new evolutionary phylogenetic assessment. Such community efforts facilitate forthcoming research in this area and ensure that newly identified enzymes from a variety of crop species will be studied appropriately.

## Emerging topics and outlook

While our knowledge of PTMs and their regulation in plants is still in its infancy, it is a rapidly growing area of research. Increasingly, PTMs are recognized as critical directors of proteome function and as major determinants of plant phenotypes. The arrival of advanced PTM-enrichment techniques, such as tandem metal oxide affinity chromatography for phosphoproteins and linkage-specific ubiquitin sensors, to name but a few (Ikeda *et al.*, 2010; Hoehenwarter *et al.*, 2013), as well as advances in quantitative proteomics and bioinformatics of PTMs, is heralding a golden age of PTM research (Ke *et al.*, 2016). While several major PTMs already enjoy widespread recognition, lesser-known modifications are now slowly gaining equally important reputations. Two examples are reviewed here: Linster and Wirtz (2018) describe the widespread role of N-terminal acetylation in stress signalling, while Serre *et al.* (2018) describe lysine methylation of proteins other than histones. Although the list of possible PTMs has grown to well over 300 (Ribet and Cossart, 2010), an even greater challenge is revealed when potential interactions between PTMs are considered. For example, numerous reports indicate direct links between phosphorylation and ubiquitination (Hunter, 2007), cross-talk between redox-based and ubiquitin or ubiquitin-like PTMs are also emerging (Skelly *et al.*, 2016), and Tomanov *et al.* (2018) predict links between SUMOylation and phosphorylation. Hence, studying all PTMs and making sense of the countless interactions between them is a major future challenge. However, the continuous development of new computational methods for delineating such big data challenges also means that the potential uses of PTM-based strategies for crop improvement are vast.
